# Supplementary material for: A qualitative study in UK secondary schools exploring how PE uniform policies influence body image attitudes and PE engagement among adolescent girls
Source: BMJ Open. 2025 Jul 17;15(7):e099312. doi: 10.1136/bmjopen-2025-099312 (PMC12273112; doi:10.1136/bmjopen-2025-099312)
Supplement: online supplemental file 2 [file bmjopen-15-7-s002.pdf]

Supplementary Table 2. Summary of PE uniform policies by school

School	PE uniform policy accessible	Discrepancies between PE uniform policy and focus groups/interviews	Unisex uniform	Options for girls (e.g. leggings)	Top colour	School had all-day PE uniform policy
1	Stated on school website	Pupils can wear branded items despite policy stating that this is not permitted<br>Pupils can wear leggings despite policy not permitting	Yes	Allowed to wear own leggings, with no strict policy on branding	White	No
2	Stated on school website	Policy states that pupils are not permitted to wear cycle shorts, however this is sometimes allowed	"No gender restrictions"	Allowed to wear own plain black sports shorts or leggings, with no branding<br>School skort was an option	White or black	No
3	Stated on school website	None	Yes	Allowed to wear plain black leggings or school branded leggings, skort and tracksuit bottoms	Black	Yes
4	Stated on school website	None	Choice between unisex or female fit for tops and shorts	Allowed to wear plain navy sports leggings or school branded leggings or tracksuit bottoms	Navy and blue	Yes
5	Stated on school website	None	Yes	Allowed to wear plain black or navy leggings with discrete branding or school branded unisex navy joggers or tracksuit bottoms	Blue	No
6	No – access only via school portal	N/A	Yes	Not allowed to wear own clothing. School-branded shorts or tracksuit bottoms given as options	White	No
