# Supplementary material for: A qualitative study in UK secondary schools exploring how PE uniform policies influence body image attitudes and PE engagement among adolescent girls
Source: BMJ Open. 2025 Jul 17;15(7):e099312. doi: 10.1136/bmjopen-2025-099312 (PMC12273112; doi:10.1136/bmjopen-2025-099312)
Supplement: online supplemental file 4 [file bmjopen-15-7-s004.pdf]

Supplementary Table 4. Benefits and considerations for schools adopting a PE uniform policy to eliminate or reduce the need to change for PE

**Allow pupils to wear PE uniform all day, on days they have PE**		**Allow pupils to come into school or leave school in their PE uniform, on days they have PE in the morning or afternoon**	
**Pros**	**Cons**	**Pros**	**Cons**
Removes feelings of discomfort, insecurity and self-consciousness when changing in front of peers in communal changing rooms, which are often described as unpleasant places	Pupils are limited to doing PE indoors when it is raining, as they may not have a change of clothing	Reduces feelings of discomfort, insecurity and self-consciousness when changing in front of peers in communal changing rooms, which are often described as unpleasant places to only once per PE class	Some pupils may try to bend the rules by wearing their PE uniform on non-PE days, which staff must monitor and sanction
Promotes inclusivity for pupils with diverse gender identities who might otherwise be expected to choose between binary changing room options	PE uniform can smell if worn all day after doing PE or if not washed on a regular enough basis	Increases time for activities during PE and breaks	Some pupils may forget or purposely not bring their school uniform to change into after PE, which staff must monitor and sanction
Increases time for activities during PE and breaks	Some pupils may try to bend the rules by wearing their PE uniform on non-PE days, which staff must monitor and sanction	Pupils are less likely to forget their PE uniform if worn into school, which reduces PE staff time spent sourcing spare uniform, and the need to sanction forgotten PE uniform	May be inconvenient to carry school uniform in a bag to change into after PE
More convenient for pupils, as they don’t need to carry their PE uniform to and around the school	Uniform may look less formal and consistent across the school	Pupils are less likely to be late to catch the bus home	Uniform may look less formal and consistent across the school

Pupils are less likely to forget their PE uniform if worn into school, which reduces PE staff time spent sourcing spare uniform, and the need to sanction forgotten PE uniform	Pupils who feel uncomfortable in their PE uniform may not have the option to change		
Pupils are less likely to be late to lessons or miss the bus home			
PE uniform is often comfier than the regular school uniform			
Belongings such as phones, jewellery, and items of clothing are less likely to go missing			
Pupils are more comfortable wearing sports bras as they don't have to change in and out of them in front of others			
