# Supplementary material for: A qualitative study in UK secondary schools exploring how PE uniform policies influence body image attitudes and PE engagement among adolescent girls
Source: BMJ Open. 2025 Jul 17;15(7):e099312. doi: 10.1136/bmjopen-2025-099312 (PMC12273112; doi:10.1136/bmjopen-2025-099312)
Supplement: Supplementary file 6 [file bmjopen-2025-099312.reviewer_comments.pdf]

PEER REVIEW HISTORY

BMJ Open publishes all reviews undertaken for accepted manuscripts. Reviewers are asked to complete a checklist review form and are provided with free text boxes to elaborate on their assessment. These free text comments are reproduced below.

ARTICLE DETAILS

Title (Provisional)

A qualitative study in UK secondary schools exploring how PE uniform policies influence body image attitudes, and PE engagement among adolescent girls

Authors

Porter, Alice; Cawley, Elin; Chapman, Laura; Crisp, Charlotte; Wadman, R.; Barber, Sally; Penton-Voak, Ian; Attwood, Angela S; Jago, Russ; Bould, Helen

---

VERSION 1 - REVIEW

**Reviewer**	**1**
**Name**	**Kerner, Charlotte**
**Affiliation**	**Brunel UNiversity London**
**Date**	**11-Apr-2025**
**COI**	**None**

Overall, this is an excellent study that makes a unique contribution to the literature and has clear practical implications to support policy and practice. The main limitation of the study is the lack of diversity in the sample, meaning, those whose voices we probably want to hear (e.g. the disengaged) were not included, however, this is a common issue of recruitment in this type of research. Whilst this is an excellent study, some work could be done around conceptualisation and links with mechanisms (see below).

I have some suggestions for developing the manuscript:

1) The authors need to more critically consider the conceptualisation of the body image construct. The authors position the definition descriptively, implying a universal definition that does not exist. This critical conceptualisation then needs to be considered throughout the manuscript e.g. when discussing findings.

2) Related to this, the authors should avoid using the terms body dissatisfaction interchangeably with body image. This leads me to question, is the study about body image or body dissatisfaction? If it is the former, this needs to be considered in the framing of the study and the discussion of the findings. E.G. body image could be multidimensional,

therefore; how were the different elements captured? If body dissatisfaction, then the definition(s) need to reflect this.

3) Throughout the manuscript, I suggest further discussion of the potential mechanisms by which the main points impact body image e.g. by linking with theory

4) I was surprised that the discussion of gender diverse identities was not framed within the introduction, given that this was part of the recruitment strategy. It would be great to see this positioned early on.

**Reviewer**	**2**
**Name**	**Nathan, Nicole**
**Affiliation**	**Population Health Hunter New England Local Health District**
**Date**	**16-Apr-2025**
**COI**	**None**

Thank you for the opportunity to review this paper. It was a pleasure to read, it's well written and addresses such an important topic. The themes identified are really relevant and deserve to be in the public domain. I have a few questions and suggestions for the authors, which I hope are helpful and can be addressed within the word limit constraints.

Background

• The background was well written and very concise. There has been more work undertaken in the primary school space about the impact of uniforms which the authors could lean on (some of which is referenced in the discussion) to help set the context for the need for this study.

• For those outside the UK it would be great to include some level of detail regarding the usual PE uniform policy/ practice i.e. do students have to get changed in and out of PE kits for sport/PE? Or can they wear them all day? What does the PE kit typically consist of is it shorts, polo shirt, trainers? Is it a "school branded whole PE kit" or can they wear own shorts etc.

Methods

• Setting- for people outside of the UK it would be helpful to give some context of the setting in which study conducted e.g. size, demographics etc. of Bristol/Gloucestershire

• Great to see the PPIE statement and that the survey was adapted based on feedback

• Data analysis and positionality- to enhance transparency it would be useful to further expand on the reflexivity/positionality statement. RTA places a strong emphasis on researcher subjectivity in the analytic process so how did your identities, interests, or beliefs

shape data interpretation? did the team engage in any reflexive practices to challenge assumptions/biases? Also the power dynamics between researchers and participants how was that managed (you could also address this in the explanation of the procedures to describe how that was managed in the focus groups).

• As the study also draws on two distinct data sources (focus groups with students and interviews with teachers) it would strengthen the analysis to provide a brief rationale for merging the datasets and clarifying how differences (or similarities) across the groups were handled during coding and theme development.

Results

• Sample- can you elaborate on why schools declined invitation? Lack of engagement of schools noted in the results but it is useful for the field to understand what the reasons are for refusal.

• Could you also give any detail if the sample of schools and the sample of participants was a good representation of the community/school? Do you have any other characteristics that you can provide for the participating schools i.e. school size, government, catholic? Rural, urban? Were you over/under represented by any demographic of participants? and if so for the discussion you may want to reflect what that means for the results.

• Theme 1- line 248-256- this is just picking up my earlier point re usual uniform practice. This suggests students can wear whatever they want in PE? This context is important to understand what is meant by PE uniforms and may have different implications in countries where a mandatory PE uniform is required.

• Co-developing PE uniform- just a suggestion, but it might be worth reminding the reader that this section draws only on data from PE staff/teachers. I found myself going back to the methods to check why this part was only focused on teachers, and not students. Also, the heading “co-developing PE uniform” feels a bit narrow for what’s covered i.e. the content seems broader, touching on what the uniform policy should include more generally (e.g. allowing PE uniforms to be worn to and from school). The inclusion of recommendations here also feels out of place. This might just be personal preference, but to me it reads more like proposed next steps than a direct presentation of the findings. I’d be inclined to move this into the discussion (or a new section after it) and position them clearly as recommendations for policy and practice- you can couch this in their limitations (ie this is based off the research team and not co-designed etc) but it seems better placed elsewhere.

Discussion

• The start of the discussion seems to simply restate the aim rather than a reflection on what the findings actually mean. I’d love to see more interpretation upfront ie what’s the key takeaway? What’s new or different from what we already know?

• Given your aims were to explore how secondary school PE uniform policies influence body image and PE engagement (participation and enjoyment) I would recommend presenting the

discussion that way. Paragraph two addresses the second aim. It also wasn't very clear to me which paragraph was addressing the first aim, is it paragraph 3 and 4 or just 3?

• Paragraph two. Opening sentence you may just want to spell out for the reader the why i.e. why these features of the uniform discourage participation. Drawing out the mechanism e.g. discomfort, embarrassment etc etc would help clarify the link between the uniform and reduced engagement in PE. This may also help (and please there is no expectation you cite this just thought this may be helpful) my colleague looked at secondary school students perspective on wearing their sports uniforms. In a sample >900 students a large majority reported that they would prefer to wear the PE uniform everyday and thought that they were more active when they wore their uniform https://www.researchgate.net/profile/Nicole-Mccarthy-2/publication/336618178_Australian_secondary_school_student%27s_attitudes_to_changing_from_traditional_school_uniforms_to_sports_uniforms/links/5da903f2299bf111d4be2270/Australian-secondary-school-students-attitudes-to-changing-from-traditional-school-uniforms-to-sports-uniforms.pdf

• Paragraph 3 I found this paragraph a bit hard to follow in terms of what's driving what. The study aims to understand how PE uniforms influence body image, but this section reports that girls with low self-esteem are already more vulnerable, and the uniform just makes things worse??. So is it the uniform causing poor body image and disengagement? Or is it that girls who already feel uncomfortable are more affected by the uniform? It starts to feel a bit circular. It also starts to refer back to this impacting participation which is covered in paragraph 1 above. I think this would be clearer if you explicitly unpacked the direction of influence even just acknowledging that it may be bidirectional. Line 468 you state 66% of girls reported high levels of enjoyment of PE. This wasn't presented in your results (I assume this is coming from your study procedures section where you asked students on their consent form). I wouldn't recommend bringing this in here, it wasn't an aim of your study ad you are inferring it from data not presented.

• Paragraph 4 seems to be closely aligned with paragraph 3- I wonder if it would be worth considering rolling this in with the above paragraph as you haven't addressed Theme 2 which I think is such an important finding, especially as schools are moving to gender neutral uniforms. It feels a shame not to explore this finding more in your discussion and the implications it may have for schools.

• Limitations depending on your reflection on the positionality I noted above there could be potential limitations of the themes developed jointly (student focus groups and teacher surveys) i.e. if the student voice is underrepresented in the themes or the integration of data sources wasn't clearly described. Similarly, as the positionality statement noted, all researchers were white, highly educated females this shared background may have shaped how data were interpreted, particularly around body image, uniform norms, and gender.

• Conclusion- many of the recommendations you have mentioned in the results are within here, which is again I think a case of bringing the recommendations down here.

**Reviewer**	**3**
**Name**	**Reidy, Johanna**
**Affiliation**	**University of Otago, Public Health**
**Date**	**21-Apr-2025**
**COI**	**None**

Thank you for undertaking this research. It is an important and neglected topic and your work documents factors that should be considered in PE uniform policy design, particularly how students feel in their PE gear.

1. **However, I would add in some extra considerations that highlight the context specificity of what you're finding and state that the process may be of interest overseas but the findings could be limited to the UK.** This work covers what people think about uniform and potentially leaves out some important elements to consider about the context. For instance overseas work focuses on environmental hazards such as UV exposure or heat and how uniform can promote safety through design. This work (eg Gage et al(Gage et al., 2017, 2018)) notes that collared shirts and slightly longer arm lengths allow for more sun protection in the harsh southern hemisphere sun. Have you given any consideration to any environmental factors in England eg uniform visibility in darkness in the winter months.
2. **The recommendations would be more impactful if you reorganised table 4**
   Your results are a bit mixed up – you've got particular garment properties (tops, bottoms, shoes etc) against questions of cost and affordability and sizing. The affordability and sizing overarch all the garments. Just for consistency perhaps put it upfront.
3. Can you be more specific about colour recommendations – is this about preferences in a colour palette or is it linked to physical safety eg temperature regulation, visibility on journey to and from school etc. You state:
   'Choose a neutral colour that is durable and can accommodate change in weather and temperature. Schools may wish to incorporate the school colour(s).' – how does colour affect weather and temperature? Are you saying white. How does your data support this recommendation?
   Round collar – does it have to be round or simply without a collar.
   You also make reference to 'well fitting' – how is this an objective definition? Some students prefer oversize as you note further down.
4. **Think about the implications of sitting in PE kit all day vs the anxiety of changing in from of one's peers.** If it's active-wear then it's going to smell pretty nasty, even

when the sweat dries. It may not have been your finding but consider addressing the option to change out of PE kit and into ordinary school uniform.

5. **Did you have any findings about the PE gear option and incidental exercise? Line 456 - Uniform design and incidental exercise –** you mention activity at break time – did you find anything about the journey to school and uniform design? (Hopkins and Mandic, 2017)?

6. **It would be interesting if you could point to the relative weightings /importance of the themes you found (if any).** I'm thinking of the uniform looking 'smart' as one of the considerations. While a PE top allows uniformity, the smartness issue is interesting**.** Is the population of students who represent their school in interschool fixtures quite large in England so that smartness is a real concern? Otherwise it looks like one of the criteria for design only affects a small number of students and how they look at 'away' fixtures – and not their actual sporting performance.

   You note that your sample is a convenience sample and that academies are more affluent than the general population.

   While I understand that one wants to get as much as possible out of a design, smartness is probably not a discriminating factor. Or does the smart uniform refer to a 'tracksuit' or full branded strip for interschool athletics or football that they wear to events? It'd be good to clarify this point.

7. **Was anything said about swimming gear**? Or is swimming not part of the curriculum in secondary school in England?

Overall this is a thoroughly interesting piece. Once again thank you for your work on this topic.

Some refs for your information.

Gage R, Leung W and et al (2017) Clothing Protection from Ultraviolet Radiation: A New Method for Assessment. *Photochemistry and Photobiology* 93(6): 1513–1518.

Gage R, Leung W, Stanley J, et al. (2018) Sun Protection Among New Zealand Primary School Children. *Health Education and Behavior* 45(5): 800–807.

Hopkins D and Mandic S (2017) Perceptions of cycling among high school students and their parents. *International Journal of Sustainable Transportation* 11(5). Taylor & Francis: 342–356.

VERSION 1 - AUTHOR RESPONSE

Reviewers' comments	Our response
**Reviewer 1**	
The authors need to more critically consider the conceptualisation of the body image construct. The authors position the definition descriptively, implying a universal definition that does not exist. This critical conceptualisation then needs to be considered throughout the manuscript e.g. when discussing findings.	We acknowledge that there is not a universal definition of body image and that the Introduction had not included our critical conceptualisation of body image.<br>We have edited the first paragraph of the Introduction to include the key conceptualisation we used to frame our study – the cognitive behavioural framework by Cash (2012). We include our chosen definition of body image and expand on how the elements of body image have been conceptualised (lines 79-88).<br>"Body image is a complex, multidimensional construct, for which there is currently no universally agreed definition. However, the cognitive behavioural framework conceptualises body image as "how people experience their own embodiment, especially, but not exclusively, their physical appearance."[5] Body image, as it relates to physical appearance, is considered to include perceptual and attitudinal elements, with body image attitudes (cognitions, emotions, and behaviours) incorporating the importance of physical appearance for self-evaluation and evaluative beliefs about physical appearance and body satisfaction.[5] Body dissatisfaction is characterized by negative cognitions and emotions regarding one's body, often stemming from a perceived discrepancy between one's own body and an 'ideal' body,[6]"<br><br>In addition, throughout the manuscript, we have changed the wording of body image to "body image attitudes" to better align with this conceptualisation, and updated the text describing our study aims to further highlight this (lines 128-130).<br>"Explore how PE uniform policies might influence body image attitudes (such as body dissatisfaction), in line with the cognitive behavioural conceptualisation of body image."
Related to this, the authors should avoid using the terms body dissatisfaction interchangeably with body image. This leads me to question, is the study about body image or body dissatisfaction? If it is the former, this needs to	In our study, we focus on body image attitudes more broadly. We conceptualise body dissatisfaction as an element of body image linking to negative evaluative beliefs about one's body. In the edits made to address the

be considered in the framing of the study and the discussion of the findings. E.G. body image could be multidimensional, therefore; how were the different elements captured? If body dissatisfaction, then the definition(s) need to reflect this.	comment above, we have hopefully now made this clearer. We feel it is important to highlight body dissatisfaction (and its definition) in the Introduction, as this is where most of the public health research has been focused, in terms of establishing links with health-related outcomes. As we describe further in our response to the following question, we have now clarified how our qualitative findings relate to specific aspects of body image attitudes, including body dissatisfaction.
Throughout the manuscript, I suggest further discussion of the potential mechanisms by which the main points impact body image e.g. by linking with theory	Throughout the Results and Discussion, we have expanded descriptions of body image to include their specific elements, rather than just referring to the term body image, to better demonstrate how our findings link with our conceptualisation of body image, and highlight the nuances and potential mechanisms. E.g.:<br>Results:<br>"3) girls look similar to their peers and do not stand out, which could, for some, exacerbate negative thoughts and feelings about their bodies (see link to Theme 3)." (lines 284-285)<br>"Psychological comfort in wearing the PE uniform appeared to be influenced by physical appearance-related concerns" (lines 422-423)<br>Discussion:<br>"PE uniforms such as those that fit poorly (e.g. unisex designs) or expose too much skin, can heighten feelings of self-consciousness, and amplify appearance-related insecurities…" (lines 495-497)<br>"Girls with low self-confidence appeared more vulnerable to the effects of uncomfortable PE uniforms on how they felt about their bodies, for example suggesting evaluative beliefs (i.e. comparing their physical appearance to others), which reduced motivation and participation in PE. Whereas, self-confident girls appeared less affected by how they would be perceived by others, and would participate regardless." (lines 500-505)
I was surprised that the discussion of gender diverse identities was not framed within the introduction, given that this was part of the recruitment strategy. It would be great to see this positioned early on.	The study predominantly aimed and was centred around exploring the views of adolescent girls, however we felt it was important to not exclude those with gender diverse identities (i.e. non-binary as described in our study). We however did not aim to purposively recruit non-binary pupils, nor did we have a sample size requirement for pupils with diverse gender identities. As this was not

	a focus or objective of the study, we do not feel it is necessary to discuss the role of gender identities in the Introduction. We have added a sentence to the Methods clarifying this (lines 161-163).
**Reviewer 2**	
Background: The background was well written and very concise. There has been more work undertaken in the primary school space about the impact of uniforms which the authors could lean on (some of which is referenced in the discussion) to help set the context for the need for this study.	We have added to paragraph 3 of the Introduction by discussing some of the literature in primary schools and highlighting the gap in secondary school research in this area (lines 113-116):<br>"There is some evidence that for younger girls, wearing an "active uniform" (a uniform that better enables physical activity) during primary school can increase physical activity,[30 31] however there is a lack of research on how physical activity enabling uniform policies may impact physical activity in secondary schools"
Background: For those outside the UK it would be great to include some level of detail regarding the usual PE uniform policy/ practice i.e. do students have to get changed in and out of PE kits for sport/PE? Or can they wear them all day? What does the PE kit typically consist of is it shorts, polo shirt, trainers? Is it a "school branded whole PE kit" or can they wear own shorts etc.	As we elude to in paragraph 4 of the Introduction, the UK government guidelines on PE uniforms for secondary schools are rather broad and vague. This means that PE uniforms are likely to vary greatly from school to school in the UK, with some schools allowing pupils to wear their PE kit all day and some that require pupils to change; similarly some schools will have several mandated branded items, whereas others might have none. This highlights the need for more specific guidance for schools, which we state here. We have added a sentence to this paragraph that highlights this likely variability in usual policy/practice (lines 122-124) (albeit there is currently no evidence on what the PE uniform policies and practices are across secondary schools in the UK, as this data is not collected at a population level).<br>"As Government guidelines in England allow secondary schools the freedom to determine their own PE uniform policies (including school branded items, colours, styles, when PE uniform can be worn),[32] these are likely to vary greatly from school to school."
Methods: Setting- for people outside of the UK it would be helpful to give some context of the setting in which study conducted e.g. size, demographics etc. of Bristol/Gloucestershire	For context for the non-UK audience, we have added some details about the population and demographics of Bristol and South Gloucestershire based on the most recent census data for the UK (2021) (lines 136-140)<br>"We conducted a qualitative study across six mixed-sex secondary schools in Bristol (urban city in South West England, approximate

	population of 472,500 (28.4% ethnic minority)) and South Gloucestershire (urban district in South West England, approximate population 290,400 (8.8% ethnic minority))[37] via focus groups with Year 8 pupils (12-13 years) and interviews with PE staff."
Methods: Great to see the PPIE statement and that the survey was adapted based on feedback	Thank you
Methods: Data analysis and positionality- to enhance transparency it would be useful to further expand on the reflexivity/positionality statement. RTA places a strong emphasis on researcher subjectivity in the analytic process so how did your identities, interests, or beliefs shape data interpretation? did the team engage in any reflexive practices to challenge assumptions/biases? Also the power dynamics between researchers and participants how was that managed (you could also address this in the explanation of the procedures to describe how that was managed in the focus groups).	We have expanded the Positionality and reflexivity section (lines 227-234). Reflexive note taking following the focus groups and interviews and during coding was part of our reflexive practice, which were then discussed during coding meetings. We also reflected and discussed our own experiences with PE uniforms when younger, which we found varied among us, which is likely to have shaped data interpretation.<br>"Following the focus groups and interviews and alongside coding, researchers made reflexive notes, which were discussed during coding meetings to consider how researcher subjectivity may shape data analysis. The researchers discussed their own varied experiences of PE uniforms when younger, and how this may also shape data interpretation."<br><br>In terms of power dynamics, we ensured that only female researchers conducted the focus groups and that the teachers were not present, to help the girls feel more comfortable talking openly about the topic. We used the interactive activities to help break the ice and to allow all pupils to have a voice, as we know that some pupils can dominate conversations and others can stay quiet, due to group dynamics. Each researcher also made reflexive notes following each focus group/interview, which included reflecting on the power dynamics (see edits above). However, we had not met the girls before so had not built up a previous rapport and the focus groups were conducted in the school setting, as logistically it would have been too difficult to conduct them elsewhere, which we appreciate may have created a hierarchical power dynamic between us as the researchers and the girls. We have edited the study procedures section to make it clearer that attempts were made to reduce the impact of power dynamics (lines 181-185) but have also added a sentence to the limitations

	highlighting this may not have been fully achieved (lines 581-586).<br>Study procedures section: “To help pupils feel as comfortable as possible and able to discuss the topic openly, focus groups were conducted by two female researchers (EC and AP or LC) without staff present. The interactive activities within the focus groups were also used to help break the ice, build rapport between researchers and pupils, and allow all pupils opportunities to voice their views.”<br><br>Limitations: “While attempts were made through our methods and reflexivity to consider and mitigate the power dynamics amongst researchers and pupils, the focus groups were conducted within the school setting, and researchers had not built up previous rapport with the pupils or met them previously to enable them to develop an understanding of their group dynamics. Hierarchy amongst the pupils, and power imbalance with the researchers, may have influenced the views they shared.”
Methods: As the study also draws on two distinct data sources (focus groups with students and interviews with teachers) it would strengthen the analysis to provide a brief rationale for merging the datasets and clarifying how differences (or similarities) across the groups were handled during coding and theme development.	We have added to the data analysis section to make it clearer why we combined the focus groups and interviews into one dataset for analysis<br>“Data from focus group and interview transcripts, and Post-it notes from pupil activities were combined to form the dataset. Data were combined to develop themes informed by the entire dataset and identifiers (pupil, staff, school) were used to allow for observation of similarities and differences within and between participants.” (lines 207-2109)<br>“When developing themes, attention was given to where staff and pupils views aligned and where they diverged to highlight the interplay between school policies and practices and pupil perspectives.” (lines 221-223)
Results: Sample- can you elaborate on why schools declined invitation? Lack of engagement of schools noted in the results but it is useful for the field to understand what the reasons are for refusal.	We have added the sentence “The majority of schools did not respond to the study invitation; those that declined the invitation reported a lack of capacity as the reason for non-participation.” (lines 241-243)
Results: Could you also give any detail if the sample of schools and the sample of participants was a good representation of the	We state in the Sample section of the Results that all schools were government funded academies (see footnote below Table 1 for

community/school? Do you have any other characteristics that you can provide for the participating schools i.e. school size, government, catholic? Rural, urban? Were you over/under represented by any demographic of participants? and if so for the discussion you may want to reflect what that means for the results.	definition of academies for non-UK context).<br>We have added further summary statistics of the participating schools to the Sample section (all schools were urban, all but one had no religious character) (lines 237-239) and Table 1 (added school size).<br>We have also added a sentence highlighting the representation of the pupils in the study compared to the wider school demographics for ethnicity and affluence, which were broadly representative – 66% of participants were of White ethnicity vs the mean percentage of pupils of White ethnicity across all schools was 67%; 77% of participants were of average or high affluence vs the mean percentage of pupils not eligible for free schools meals (measure of affluence) was 78%.<br>“The majority of pupils were of White ethnicity (66%) and from families who perceived to earn average or above average income (77%), which was representative of the wider school demographics, as shown in Table 1.” (lines 256-259)<br>We are not able to compare the sample of schools to the wider school demographics of the study areas, as this would require additional quantitative analysis of the UK government school database, which we do not feel is necessary for this study. However, we have added a sentence to the Limitations to highlight that our sample of schools may not have been representative of the wider population of schools in the study area (lines 580-581)<br>“also resulting in a sample which may not have been representative of the study areas.”
Results: Theme 1- line 248-256- this is just picking up my earlier point re usual uniform practice. This suggests students can wear whatever they want in PE? This context is important to understand what is meant by PE uniforms and may have different implications in countries where a mandatory PE uniform is required.	In the UK context, secondary school pupils are usually required to wear a mandated school PE uniform and not usually allowed to wear whatever they want. However this is hugely variable across schools and some schools for example may require pupils to wear a certain school-branded top but allow them to wear their own bottoms in a particular colour vs other schools may mandate the whole PE uniform. However, the girls expressed that they would prefer to wear whatever they wanted, as this would make them feel more comfortable, which is what we describe within Theme 1. Supplementary Table 2 describes the PE uniform for each school and highlights the

	variability across schools, which we refer to on line 262 but we have also added a sentence making it clearer that all schools in the sample required pupils to wear a mandated school PE uniform in some form (line 264-265), which should help to set the findings within context. “All schools in the sample required pupils to wear a mandated school PE uniform.”
Results: Co-developing PE uniform- just a suggestion, but it might be worth reminding the reader that this section draws only on data from PE staff/teachers. I found myself going back to the methods to check why this part was only focused on teachers, and not students. Also, the heading “co-developing PE uniform” feels a bit narrow for what’s covered i.e. the content seems broader, touching on what the uniform policy should include more generally (e.g. allowing PE uniforms to be worn to and from school). The inclusion of recommendations here also feels out of place. This might just be personal preference, but to me it reads more like proposed next steps than a direct presentation of the findings. I’d be inclined to move this into the discussion (or a new section after it) and position them clearly as recommendations for policy and practice- you can couch this in their limitations (ie this is based off the research team and not co-designed etc) but it seems better placed elsewhere.	Although this section draws mainly from the staff interviews because it is about the logistics and practical implications of changing PE uniform polices, we did also include data from pupils where relevant. This is shown in one of the quotes (line 468 is a quote from a pupil) and where we summarise the data in Supplementary Table 5.<br>We have changed the sub-heading to “Practical implications of changing PE uniform policies in secondary schools” and have moved Table 4 to the Discussion, where we have tied it into the Implications for policy and future work section (lines 554-556).<br>“Informed by our findings, Table 4 provides initial recommendations for improving secondary school PE uniform policies for adolescent girls.”<br><br>We have also therefore added to the second paragraph of the Practical implications results section by highlighting some of the key changes suggested by pupils and staff with supporting quotes (lines 446-459), which are presented in full in Supplementary Table 3 and then summarised in our conclusions and Table 4 in the Discussion.
Discussion: The start of the discussion seems to simply restate the aim rather than a reflection on what the findings actually mean. I’d love to see more interpretation upfront ie what’s the key takeaway? What’s new or different from what we already know?	We have expanded the first paragraph of the discussion to include the key findings (lines 489-494).<br>“This qualitative study with Year 8 pupils and PE staff highlighted how PE uniforms can negatively influence body image attitudes and PE engagement among adolescent girls, through uncomfortable clothing that limits movement. The study highlights the potential benefits of allowing pupils to wear PE uniforms all day to increase physical activity and reduce negative body image concerns associated with changing, and offers initial recommendations for redesigning PE uniform policies in UK secondary schools to improve girls’ experiences.”

Discussion: Given your aims were to explore how secondary school PE uniform policies influence body image and PE engagement (participation and enjoyment) I would recommend presenting the discussion that way. Paragraph two addresses the second aim. It also wasn't very clear to me which paragraph was addressing the first aim, is it paragraph 3 and 4 or just 3?	We have restructured the Discussion, so that the paragraphs discussing the body image findings and literature come first, followed by the PE engagement findings and physical activity literature.<br><br>Paragraphs 3 and 4 address findings relating to body image, which we have combined and restructured following your suggestion in a comment below.
Discussion: Paragraph two. Opening sentence you may just want to spell out for the reader the why i.e. why these features of the uniform discourage participation. Drawing out the mechanism e.g. discomfort, embarrassment etc etc would help clarify the link between the uniform and reduced engagement in PE. This may also help (and please there is no expectation you cite this just thought this may be helpful) my colleague looked at secondary school students perspective on wearing their sports uniforms. In a sample >900 students a large majority reported that they would prefer to wear the PE uniform everyday and thought that they were more active when they wore their uniform	We have added more detail to this sentence, highlighting the mechanisms we found (lines 531-534).<br>"Our findings highlighted that PE uniforms that fit poorly, are too short, restrictive, and not weather appropriate (e.g. short skorts, tight collars, thin jackets) can discourage PE participation due to limiting movement, feeling too self-conscious, and either too cold or too hot to participate fully."<br><br>This is a really relevant study for our discussion, which we have included on lines 544-548 in combination with another relevant study highlighted by another reviewer.<br>"In addition, qualitative research with adolescent girls in New Zealand found that not having an activity-enabling uniform was a barrier to cycling to school,[55] and in a large survey, 72% of Australian adolescent girls reported that they would prefer to wear their sports uniform every day, with 65% believing this would increase their physical activity during school.[56]"
Discussion: Paragraph 3 I found this paragraph a bit hard to follow in terms of what's driving what. The study aims to understand how PE uniforms influence body image, but this section reports that girls with low self-esteem are already more vulnerable, and the uniform just makes things worse??. So is it the uniform causing poor body image and disengagement? Or is it that girls who already feel uncomfortable are more affected by the uniform? It starts to feel a bit circular. It also starts to refer back to this impacting participation which is covered in paragraph 1 above. I think this would be clearer if you explicitly unpacked the direction of influence even just acknowledging that it may be bidirectional. Line 468 you state 66% of girls	Our findings do suggest a bidirectional relationship whereby an uncomfortable ill-fitting PE uniform can reduce self-confidence but also high self-confidence can be protective against PE uniform making girls feel self-conscious. We refer to participation in this paragraph because the theme included how PE uniform is linked to self-confidence and in turn PE engagement, so feel it is relevant to discuss it in this paragraph as well, as we aimed to explore the link between body image and PE engagement. We have restructured and edited the text to firstly discuss the body image and self-confidence findings, followed by the impact on PE engagement, and have more clearly highlighted how we have interpreted

reported high levels of enjoyment of PE. This wasn't presented in your results (I assume this is coming from your study procedures section where you asked students on their consent form). I wouldn't recommend bringing this in here, it wasn't an aim of your study ad you are inferring it from data not presented.	the influence of self-confidence (lines 495-505).<br>"Our findings suggested that uncomfortable PE uniforms such as those that fit poorly (e.g. unisex designs) or expose too much skin, can heighten feelings of self-consciousness, and amplify appearance-related insecurities, such as those in relation to body hair, and shape, as can communal changing rooms. These insecurities may negatively impact PE engagement. Self-confidence appeared to play an important role in the relationship between girls' views about their PE uniform and their body image attitudes. Girls with low self-confidence appeared more vulnerable to the effects of uncomfortable PE uniforms on how they felt about their bodies, for example suggesting evaluative beliefs (i.e. comparing their physical appearance to others), which reduced motivation and participation in PE. Whereas, self-confident girls appeared less affected by how they would be perceived by others, and would participate regardless."<br><br>PE enjoyment was collected as part of the pupil characteristics and is presented in the Results section in Table 2, however we have now removed this text after restructuring.
Discussion: Paragraph 4 seems to be closely aligned with paragraph 3- I wonder if it would be worth considering rolling this in with the above paragraph as you haven't addressed Theme 2 which I think is such an important finding, especially as schools are moving to gender neutral uniforms. It feels a shame not to explore this finding more in your discussion and the implications it may have for schools.	We have restructured the Discussion and combined these two paragraphs (lines 495-520).<br><br>We have added a paragraph discussing the findings on unisex uniforms and its implications for schools (lines 521-530)<br>"Our findings showed that although PE staff perceived having one unisex PE uniform for all pupils may be more practical and inclusive, many girls disliked unisex clothing, as they felt the style and fit was tailored for male rather than female bodies, and could contribute to feelings of discomfort. Therefore, providing several non-gendered options for style and fit was suggested to reduce discomfort, whilst also promoting gender inclusivity. This highlights the nuances of gendered sportswear that schools need to consider when developing PE uniform policies. In addition, our findings aligns with research drawing from young women's experiences of PE uniforms, which highlight the need for gender-neutral uniforms

	that reduce gender differences when playing sport but also fit female body shapes to better enable participation.[29]"
Discussion: Limitations depending on your reflection on the positionality I noted above there could be potential limitations of the themes developed jointly (student focus groups and teacher surveys) i.e. if the student voice is underrepresented in the themes or the integration of data sources wasn't clearly described. Similarly, as the positionality statement noted, all researchers were white, highly educated females this shared background may have shaped how data were interpreted, particularly around body image, uniform norms, and gender.	We have addressed this comment above and added an additional sentence to the limitations regarding positionality (lines 586-587)<br>"In addition, the positionality of the researchers analysing the data (all White, highly educated females) may have shaped data interpretation."
Discussion: Conclusion- many of the recommendations you have mentioned in the results are within here, which is again I think a case of bringing the recommendations down here.	We have addressed this comment above.
**Reviewer 3**	
I would add in some extra considerations that highlight the context specificity of what you're finding and state that the process may be of interest overseas but the findings could be limited to the UK. This work covers what people think about uniform and potentially leaves out some important elements to consider about the context. For instance overseas work focuses on environmental hazards such as UV exposure or heat and how uniform can promote safety through design. This work (eg Gage et al(Gage et al., 2017, 2018)) notes that collared shirts and slightly longer arm lengths allow for more sun protection in the harsh southern hemisphere sun. Have you given any consideration to any environmental factors in England eg uniform visibility in darkness in the winter months.	This is a valuable point. We did not specifically explore the environmental factors, nor were they raised in the interviews and focus groups, which may reflect the less extreme weather conditions in the UK – the main consideration was for tops to reflect the heat in summer but this was mainly from a feeling hot and sweaty perspective rather than a UV protection perspective. We are therefore not able to comment on this in our findings but have included this as a consideration for future work in the Discussion (lines 564-566) and in the limitations section (line 592-593).<br>"It is also important to consider the environmental safety of redesigning PE uniforms to ensure pupils are protected from excess UV exposure and heat, as well as being visible in the dark.[57]"<br>"Further research is required to ensure these recommendations are inclusive for all pupils, including those with different gender identities, Special Education Needs and Disabilities (SEND) and from ethnic minority backgrounds, as well as environmentally safe (e.g. sun exposure)."
The recommendations would be more impactful if you reorganised table 4 Your results are a bit mixed up – you've got particular garment properties (tops, bottoms, shoes etc) against	We have moved sizing and affordability to the top of Table 4 (and Supplementary Table 3).

questions of cost and affordability and sizing. The affordability and sizing overarch all the garments. Just for consistency perhaps put it upfront.	
Can you be more specific about colour recommendations – is this about preferences in a colour palette or is it linked to physical safety eg temperature regulation, visibility on journey to and from school etc. You state: 'Choose a neutral colour that is durable and can accommodate change in weather and temperature. Schools may wish to incorporate the school colour(s).' – how does colour affect weather and temperature? Are you saying white. How does your data support this recommendation? Round collar – does it have to be round or simply without a collar. You also make reference to 'well fitting' – how is this an objective definition? Some students prefer oversize as you note further down.	In terms of colour, this detail is included within Supplementary Table 3, where we expand in detail about each of the recommendations and provide the supporting evidence. However, as this is an important point, we have added this detail to Table 4 also.<br>"Avoid black in the summer as this can be too hot. Avoid white as this can be see-through." (Table 4, line 571)<br>The recommendation for a well-fitting, round neck top comes from the data suggesting girls dislike when the collar is too tight or baggy (i.e. doesn't fit well) and V-neck tops as these can feel too low. We include this detail alongside the supporting quotes in Supplementary Table 3. We have changed the wording to be more specific to "Choose a round neck t-shirt without a collar that is not too tight nor too baggy around the neck." (Table 4, line 571)
Think about the implications of sitting in PE kit all day vs the anxiety of changing in from of one's peers. If it's active-wear then it's going to smell pretty nasty, even when the sweat dries. It may not have been your finding but consider addressing the option to change out of PE kit and into ordinary school uniform.	The advantages and disadvantages of having an all-day PE uniform policy are presented in Supplementary Table 4, which includes this consideration of the PE uniform smelling.<br>Our recommendation for the all-day PE uniform policy in the Implication section suggests allowing pupils to stay in their uniform if they wish to rather than mandating that they do.
Did you have any findings about the PE gear option and incidental exercise? Line 456 - Uniform design and incidental exercise – you mention activity at break time – did you find anything about the journey to school and uniform design? (Hopkins and Mandic, 2017)?	We don't have any findings on the impact of PE uniforms on physical activity outside of PE lessons but this is likely because our topic guide mainly focused on PE lessons. The only relevant finding we have related to the journey to/from school is that being allowed to wear their PE uniform home (i.e. not having to change) may help to avoid missing the bus home, which we include in Supplementary Table 4.<br>Nevertheless, this point and reference is very relevant to our discussion so we have added this here (lines 544-546 and 548-551).<br>"In addition, qualitative evidence with adolescent girls in New Zealand found that not having an activity-enabling uniform was a barrier to cycling to school,[55]…"<br>"Further research is required to determine whether redesigning secondary school PE

	uniforms can improve girls' PE engagement and incidental activity outside of PE lessons (e.g. at breaktimes, travelling to school)."
It would be interesting if you could point to the relative weightings /importance of the themes you found (if any). I'm thinking of the uniform looking 'smart' as one of the considerations. While a PE top allows uniformity, the smartness issue is interesting. Is the population of students who represent their school in interschool fixtures quite large in England so that smartness is a real concern? Otherwise it looks like one of the criteria for design only affects a small number of students and how they look at 'away' fixtures – and not their actual sporting performance. You note that your sample is a convenience sample and that academies are more affluent than the general population. While I understand that one wants to get as much as possible out of a design, smartness is probably not a discriminating factor. Or does the smart uniform refer to a 'tracksuit' or full branded strip for interschool athletics or football that they wear to events? It'd be good to clarify this point.	This is a good point about smartness. We did not collect data on the participating pupils' engagement with extracurricular sports clubs or fixtures but this did come up a bit in the focus groups, and as we state in the results, the sample was mainly made up of girls who reported to enjoy PE, and therefore probably more likely to be involved in fixtures etc. Our findings were weighted more towards comfort being the most important factor for the girls, but PE staff valued smartness, which is why the theme refers to the challenge of balancing both. We have edited Theme 1 in the Results to better highlight this point.<br>"Feeling comfortable in the PE uniform was most important to Year 8 girls." (line 288)<br>"Although pupils appeared to value comfort over smartness, some involved in competitions and extracurricular sports highlighted that they would prefer a smarter uniform to stand out at fixtures against other schools." (lines 341-343)<br>"when you're doing competitions and stuff it's just we look like a bunch of other schools… I would also like a different top for the competitions" (Pupils, School 6) (lines 353-354)<br><br>Just to note, academy schools in the UK are not necessarily more affluent than local authority schools but our convenience sample did include mostly affluent schools (based on government-level data on the demographics of the school community).
Was anything said about swimming gear? Or is swimming not part of the curriculum in secondary school in England?	Swimming is not part of the national curriculum in secondary schools in the UK.
Some refs for your information. Gage R, Leung W and et al (2017) Clothing Protection from Ultraviolet Radiation: A New Method for Assessment. Photochemistry and Photobiology 93(6): 1513–1518. Gage R, Leung W, Stanley J, et al. (2018) Sun Protection Among New Zealand Primary School Children. Health Education and Behavior 45(5): 800–807. Hopkins D and Mandic S (2017) Perceptions of cycling among high school students and their parents. International Journal of Sustainable Transportation 11(5). Taylor & Francis: 342– 356	Thank you for these helpful references.

VERSION 2 - REVIEW

**Reviewer**	**2**
**Name**	**Nathan, Nicole**
**Affiliation**	**Population Health Hunter New England Local Health District**
**Date**	**09-Jun-2025**
**COI**	

Thank you, the authors have addressed all comments thoroughly. Congratulations this will make an important contribution to the literature
